# Optimising case detection within UK electronic health records: use of multiple linked databases for detecting liver injury

**DOI:** 10.1136/bmjopen-2016-012102

**Published:** 2016-09-01

**Authors:** Kevin Wing, Krishnan Bhaskaran, Liam Smeeth, Tjeerd P van Staa, Olaf H Klungel, Robert F Reynolds, Ian Douglas

**Affiliations:** 1 Department of Non-communicable Disease Epidemiology, London School of Hygiene and Tropical Medicine, London, UK; 2 Department of Pharmacoepidemiology, Utrecht Institute for Pharmaceutical Sciences (UIPS), Utrecht University, Utrecht, The Netherlands; 3 Health eResearch Centre, University of Manchester, Manchester, UK; 4 Department of Epidemiology, Pfizer, New York, New York, USA

**Keywords:** PRIMARY CARE, STATISTICS & RESEARCH METHODS

## Abstract

**Objectives:**

We aimed to create a ‘multidatabase’ algorithm for identification of cholestatic liver injury using multiple linked UK databases, before (1) assessing the improvement in case ascertainment compared to using a single database and (2) developing a new single-database case-definition algorithm, validated against the multidatabase algorithm.

**Design:**

Method development for case ascertainment.

**Setting:**

Three UK population-based electronic health record databases: the UK Clinical Practice Research Datalink (CPRD), the UK Hospital Episodes Statistics (HES) database and the UK Office of National Statistics (ONS) mortality database.

**Participants:**

16 040 people over the age of 18 years with linked CPRD–HES records indicating potential cholestatic liver injury between 1 January 2000 and 1 January 2013.

**Primary outcome measures:**

(1) The number of cases of cholestatic liver injury detected by the multidatabase algorithm. (2) The relative contribution of each data source to multidatabase case status. (3) The ability of the new single-database algorithm to discriminate multidatabase algorithm case status.

**Results:**

Within the multidatabase case identification algorithm, 4033 of 16 040 potential cases (25%) were identified as definite cases based on CPRD data. HES data allowed possible cases to be discriminated from unlikely cases (947 of 16 040, 6%), but only facilitated identification of 1 definite case. ONS data did not contribute to case definition. The new single-database (CPRD-only) algorithm had a very good ability to discriminate multidatabase case status (area under the receiver operator characteristic curve 0.95).

**Conclusions:**

CPRD–HES–ONS linkage confers minimal improvement in cholestatic liver injury case ascertainment compared to using CPRD data alone, and a multidatabase algorithm provides little additional information for validation of a CPRD-only algorithm. The availability of laboratory test results within CPRD but not HES means that algorithms based on CPRD–HES-linked data may not always be merited for studies of liver injury, or for other outcomes relying primarily on laboratory test results.

**Strengths and limitations of this study**

- Development of a new clearly defined and reproducible algorithm for the detection of liver injury using linked UK electronic health record databases.
- Development of a primary care algorithm capitalising on the strength of the linked data algorithm but usable on any group of primary care patients, irrespective of linkage status.
- A lack of accessible laboratory test result data in UK secondary care electronic health records limits the added value of UK secondary care data for detecting liver injury.

## Background

Electronic health records stored within very large population-based primary and secondary care databases are an increasingly important research resource internationally. These are longitudinal records, capturing information generated as part of routine clinical care.^1^ A record for an individual patient will include anonymised information on demographics, diagnoses, prescriptions and referrals. Epidemiological studies within these databases may apply case-identification algorithms to identify disease cases that may have occurred months or years previously, for inclusion in (historical) cohort or case–control analyses.^2–4^ An alternative approach involves active case detection, continuously screening the databases so that cases may be selected for inclusion in analysis as they arise in the source population.^5^

Critical for epidemiological studies and active case detection is the ability to accurately identify outcomes. This is often challenging within these databases, where the information has been entered as part of routine clinical care, and not for the purpose of a specific study. Outcome definitions can therefore only be based on the information recorded as part of this care, which may be non-specific, and/or challenging to distinguish from all the other healthcare information recorded for that patient. Furthermore, individual databases cover a single care setting (eg, primary or secondary care), meaning that ascertainment of case status may be based on data that are only a partial description of overall healthcare.

In recent years, data linkages have been created between databases. This provides the potential to perform case ascertainment using a richer and more detailed set of data than in a single database, as information from multiple healthcare settings can be combined. To ensure that analyses do not have to be limited only to the subset of patients who have records present in each of the linked databases (reducing power for epidemiological studies and making active case detection very slow), there is the potential to use an algorithm developed using data from multiple linked databases to validate a single-database algorithm. This single-database algorithm could then be applied to the entire population of that database, irrespective of linkage status.

In this study, we focused on cholestatic liver injury, a subtype of serious liver injury and a common reason for drug licence withdrawal.^6^ ^7^ Our main aim was to test whether a multidatabase cholestatic liver injury case-identification algorithm that used linked UK databases would allow improved case ascertainment, compared to using an unlinked primary care database. A secondary aim was then to use the multidatabase algorithm to validate a new single-database primary care algorithm that capitalised on the strength of the linked data but could be used on any patient within the primary care database, irrespective of whether the individual had records linked to other databases or not.

## Methods

### Study aim

The primary aim was to assess whether a ‘multidatabase’ cholestatic liver injury case-identification algorithm that used linked UK databases would allow improved case ascertainment, compared to using an unlinked primary care database. A secondary aim was then to use the multidatabase algorithm to validate a new Clinical Practice Research Datalink (CPRD)-only algorithm that capitalised on the strength of the linked data but could be used on any patient within the CPRD database, irrespective of whether they had records linked to other databases or not.

### Setting/data sources

Three linked UK databases were used in this study: the primary care CPRD, the secondary care Hospital Episodes Statistics (HES) database and the Office of National Statistics (ONS) mortality database.^8^ Data were extracted covering the dates of 1 January 2000 to 1 January 2013. Further information is provided in the online supplementary material, 1. Description of data sources.

10.1136/bmjopen-2016-012102.supp1 supplementary material

### Outcome

The outcome was cholestatic liver injury, characterised by symptoms, including jaundice, a distinct pattern of liver test results and hospital procedure results.^9^ Cholestatic liver injury has the potential to be well ascertained by using a combination of primary and secondary care data, as an individual's care is likely to include symptoms, tests, procedures and diagnoses performed across care settings.

### Algorithm development

Two algorithms were developed for the identification of cholestatic liver injury; one multidatabase algorithm using CPRD, HES and ONS data and a second CPRD-only algorithm using data only from within the CPRD database (validated against the multidatabase algorithm).

### Multidatabase cholestatic liver injury algorithm development

**Figure 1:**
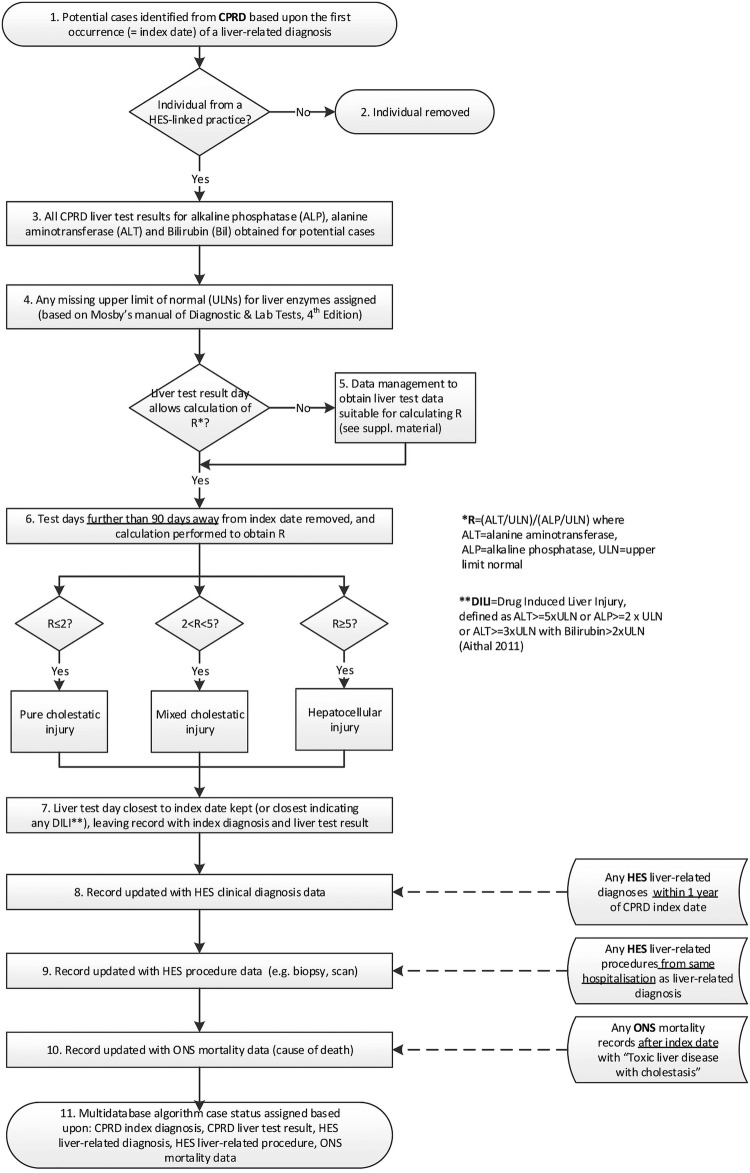
Overview of steps performed by the multidatabase cholestatic liver injury algorithm. CPRD, Clinical Practice Research Datalink; HES, Hospital Episodes Statistics; ONS, Office of National Statistics.

Multidatabase algorithm development was facilitated by reviewing studies selected by a systematic literature search as detailed in the online supplementary material, 2. Literature search for multidatabase algorithm development,^2^ ^4^ ^10–19^ along with a recent study on ascertainment of liver injury in two primary care databases^3^ and a paper describing an international consensus meeting on drug-induced liver injury.^9^ Diagnostic terms, codelists and laboratory parameters were selected based on a review of all these papers, with final terms and overall algorithm design reviewed by a member of the study team who is a general practitioner and professor in clinical epidemiology (LS). Figure 1 provides an overview of the design of the multidatabase cholestatic liver injury algorithm, detailing the steps performed in order to assign a cholestatic liver injury case status.

#### Selection of participants from CPRD (primary care) records based on liver diagnostic codes

A list of clinical diagnostic Read codes that could represent cholestatic liver injury was prepared using the search terms detailed in the online supplementary material. These were separated into three groups according to strength of evidence for liver injury, with group 1 including only the term ‘toxic liver disease with cholestasis’, group 2 consisting of jaundice-related terms and group 3 including other less specific liver pathology terms (see online supplementary material, 3. Diagnostic terms indicating liver injury for the full list of terms/codes and their grouping, also uploaded to the public ClinicalCodes.org repository^20^). The CPRD database was searched for individuals over the age of 18 years with a first occurrence of any of the liver-related terms between 1 January 2000 and 31 January 2013 who had at least 12 months follow-up prior to their index (diagnosis) date (in order to ensure the reliability of any diagnoses). Any individuals from practices that were not linked to HES were then removed (ie, practices in Scotland or Wales).

#### Management of CPRD liver test data

All CPRD test results for bilirubin, alkaline phosphatase (ALP) and alanine aminotransferase (ALT) were selected for the cohort. Blood levels of these enzymes are standard parameters for indicating and classifying serious liver injury based on the R value (=the ratio of (ALT/ULN)/(ALP/ULN), where the ULN is the upper limit of the normal blood level for the enzyme).^9^ Details of the classification and data management/cleaning performed to obtain R values are provided in the online supplementary material, 4. Classification and data management of test results. Following classification, any results >90 days from the index diagnosis date were then removed.

#### Selection of HES diagnoses and procedures and ONS mortality data

The same search terms used previously were used to search the HES diagnostic terms (coded according to ICD-10). Hospital diagnoses were considered to be more accurate than primary care diagnoses, due to specialised clinical care and the availability of additional procedures, so only two relatively specific terms were selected: ‘toxic liver disease with cholestasis’ (group 1) and ‘unspecified jaundice’ (group 2) (see online supplementary material, 3. Diagnostic terms indicating liver injury). Liver-related procedures (such as a biopsy or a scan) can support the classification of the type of liver injury,^9^ and a list of relevant procedure terms was prepared (see online supplementary material, 5. HES procedure terms). HES hospital diagnosis data for the cohort were then searched for (1) any liver-related diagnosis within 1 year before or after the CPRD index date and (2) any liver-related procedure performed during the same hospitalisation as any liver-related diagnosis.

**Figure 2:**
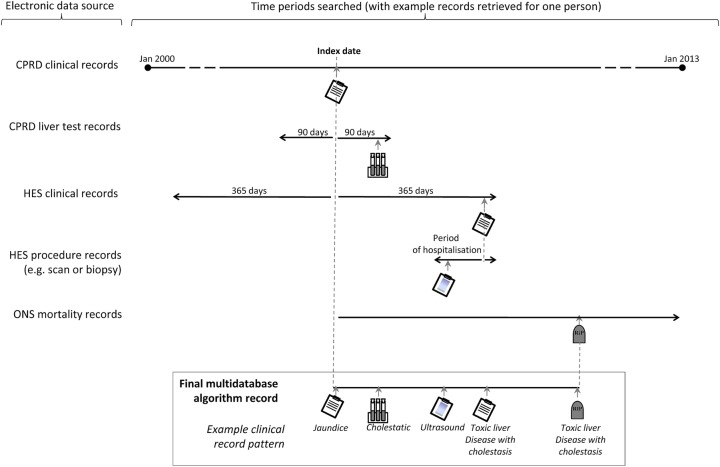
Data sources and time periods searched in obtaining data for a multidatabase algorithm cholestatic liver injury health record. CPRD, Clinical Practice Research Datalink; HES, Hospital Episodes Statistics; ONS, Office of National Statistics.

Figure 2 provides an overview of all the data sources and time periods searched in obtaining data for a multidatabase algorithm cholestatic liver injury health record.

#### Multidatabase algorithm status assignment

The multidatabase cholestatic liver injury algorithm case status was then assigned based on the presence or absence of data from each of the databases. Anyone with a CPRD cholestatic liver test result was considered to be a ‘definite’ case, based on internationally agreed consensus of the importance of biochemical criteria.^9^ ^21^ Individuals who had died and had an ONS ICD code that indicated a death certificate coded with ‘toxic liver disease with cholestasis’ (group 1) were also considered to be definite cases, in addition to individuals who were assigned this code in hospital after a biopsy or scan. Subsequent case statuses (from very likely through probable, possible, least likely and non-case) were then assigned as detailed in table 1.

**Table 1:** Assignment of the multidatabase algorithm case status using CPRD, HES and ONS data

Serial number	CPRD (Read) diagnostic code	HES diagnostic (ICD-10) code (plus HES procedural code and ONS mortality code, where considered)	CPRD liver test result	Multidatabase algorithm case status
1.	Group 1|2|3*	Not considered	Cholestatic	Definite
2.	Group 1|2|3	ONS (death): group 1	Not considered	Definite
3.	Group 1|2|3	Biopsy/scan+group 1	Not considered	Definite
4.	Group 1	Group 1	None†	Very likely
5.	Group 1	Group 2 or no HES record‡	None	Probable
6.	Group 2|3	Group 1	None	Probable
7.	Group 1	Group 1|2	Not cholestatic§	Possible
8.	Group 1	HES record has no codes of interest¶	None	Possible
9.	Group 2	Group 1	Not cholestatic	Possible
10.	Group 2	Group 2	None	Possible
11.	Group 1	No HES record|HES record has no codes of interest	Not cholestatic	Least likely
12.	Group 2	No HES record|HES record has no codes of interest	None	Least likely
13.	Group 3	Group 1	Not cholestatic	Least likely
14.	Group 2	Group 2|no HES record|HES record has no codes of interest	Not cholestatic	Non-case
15.	Group 3	Group 2	None|not cholestatic	Non-case
16.	Group 3	No HES record	None|not cholestatic	Non-case
17.	Group 3	HES record has no codes of interest	None|not cholestatic	Non-case

*Group 1: toxic liver disease with cholestasis, group 2: jaundice-related codes and group 3: other less specific liver injury codes (see online supplementary data, 3. Diagnostic terms indicating liver injury for full lists of terms).

†No liver test result recorded within 90 days of index diagnosis.

‡No HES record indicates person did not attend hospital <1 year either side of index diagnosis.

§Liver test result was recorded <90 days from index diagnosis, but results indicate either no injury or pure hepatic injury.

¶Person attended hospital <1 year from index diagnosis but no liver diagnoses of interest.

CPRD, Clinical Practice Research Datalink; HES, Hospital Episodes Statistics; ONS, Office of National Statistics.

### CPRD cholestatic liver injury algorithm development

#### Selection of participants and setup of explanatory/response variables

The CPRD database was searched for individuals over the age of 18 years with a first occurrence of any of the liver-related codes between 1 January 2000 and 31 January 2013 who had at least 12 months follow-up prior to their index diagnosis date (index date), to ensure that only incident cases were assessed. Binary variables (0, 1) were then created for each potential explanatory variable. Four main characteristics were considered a priori to be potential predictors of the multidatabase algorithm cholestatic liver injury case status: liver test result information, hospital referral information around the index date, the type of liver-related index diagnosis and information on any other liver-related diagnosis apart from the index diagnosis. A full list of the potential explanatory variables considered is provided in the online supplementary material, 6. List of CPRD algorithm explanatory variables. The outcome (response variable) was the multidatabase case status, categorised so that a value of 1 was a multidatabase case status of definite through to possible, while 0 was a multidatabase case status of least likely or non-case.

#### Statistical analysis

The cohort was randomly split into two separate data sets of equal size, one for statistical model building (the training data set) and the other for testing of the model (the validation data set).

Using the training data set, the potential CPRD explanatory variables were tabulated against case status. CPRD explanatory variables that perfectly predicted multidatabase definite-possible case status (ie, 100% of the individuals in one of the binary categories of the potential explanatory variable were cases) were removed from subsequent univariable and multivariable analysis, as were any variables with zero individuals within any category. Univariable analysis was then performed.

In multivariable analysis, Firth's logistic regression methodology was used, which can handle strata with sparse data by using penalised maximum likelihood estimation.^22^ An initial multivariable logistic regression model was fitted that included all potential CPRD explanatory variables. A final CPRD algorithm model was then prepared by removing variables with p>0.05 from the fully adjusted model in a stepwise fashion, in order of increasing strength of evidence for association. Likelihood ratio tests were used to obtain p values.

STATA (V.14.1) was used for all statistical analysis.

#### CPRD algorithm score generation and assignment

Variables for storing explanatory variable ‘scores’ were added to the validation data set, and if an individual had a value of 1 for any of the CPRD explanatory variables, the corresponding score variable was populated with the multivariable regression analysis log odds value. Those individuals who had a ‘1’ for any of the variables shown to be perfect predictors of multidatabase case status were assigned a ‘perfect prediction’ CPRD algorithm score (a score that was manually inputted as higher than the highest combined possible explanatory variable score). A total score variable was created to hold the total score for an individual, based on the presence of CPRD explanatory variables.

#### Receiver operator characteristic analysis of CPRD cholestatic liver injury algorithm and consideration of cut-off scores

The ability of the CPRD cholestatic liver injury algorithm to discriminate between the two multidatabase cases statuses (definite to possible vs least likely to non-) was assessed by plotting a receiver operator characteristic (ROC) graph (sensitivity vs 1−specificity) across the range of CPRD algorithm scores.

## Results

### Participants

**Figure 3:**
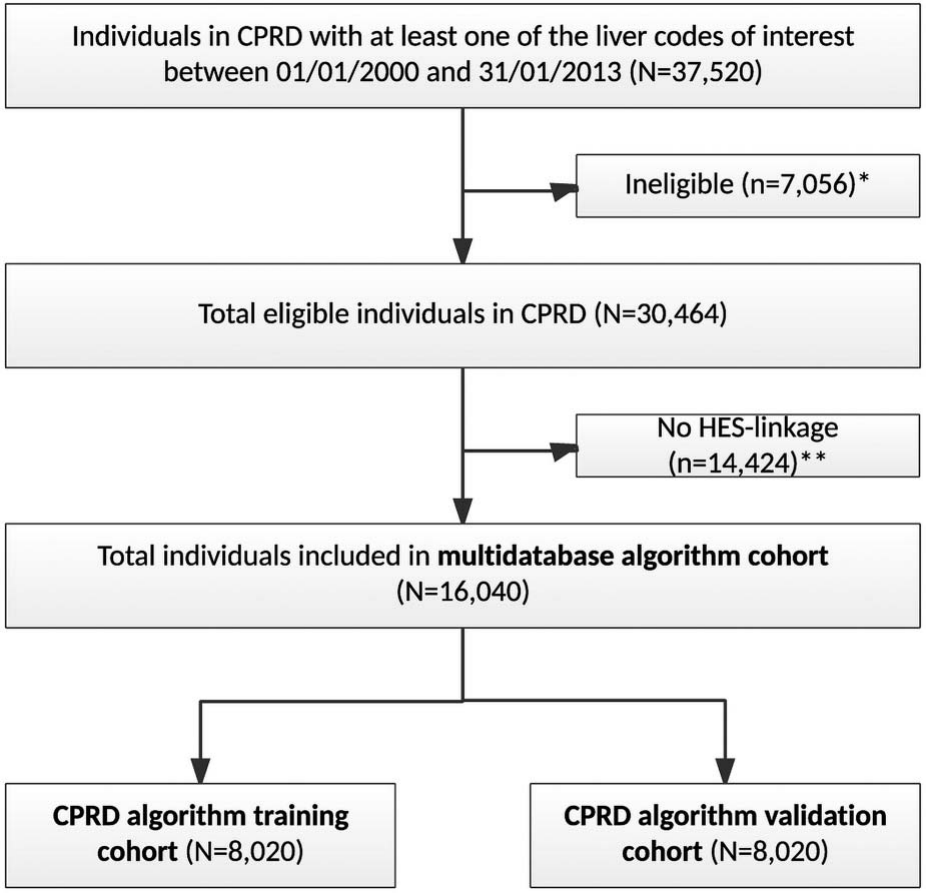
Flow of number of individuals included in the multisource algorithm and the Clinical Practice Research Datalink (CPRD) algorithm cohorts. *Ineligible: <18 years of age or registered in CPRD for <12 months prior to liver-related diagnosis. **No Hospital Episodes Statistics (HES) linkage: individual was registered with a primary care practice that was not part of the HES-linkage process (ie, practices in Scotland, Wales or Northern Ireland; English practices that have not agreed to participate and patients within participating practices that have opted out).

Between 1 January 2000 and 1 January 2013, 37 520 people were identified in CPRD with codes indicative of possible liver injury. Seven thousand and fifty-six people were removed as they were ineligible (see figure 3), and removal of a further 14 424 individuals from practices not linked to HES left a total of 16 040 individuals in the multidatabase algorithm cohort. Dividing this randomly into two data sets left 8020 people in the CPRD-only algorithm training and validation cohorts.

### Multidatabase algorithm development

#### Descriptive data

The median age of the cohort was 62 years, and 52% were men (table 2). There was a slight increase in the number of diagnoses for the codes of interests over the recruitment period of 2000–2012 (with 30% of codes diagnosed between 2009 and 2012), but only in accordance with the increase in size of the database between 2009 and 2012 (see online supplementary data, 7. Increase in size of CPRD database from 2000 onwards). The most common CPRD index diagnoses codes were jaundice, obstructive jaundice and cholangitis. Over 54% of people had a CPRD liver test result recorded within 90 days of their index diagnosis date. Seventy-nine per cent of people had been admitted to hospital for any reason within 1 year either side of the index diagnosis date.

**Table 2:** Characteristics of people (N=16 040) included in the multidatabase algorithm cohort (data from CPRD record unless otherwise stated)

	n (%)
Age at index diagnosis date*	
18–29	948 (6)
30–39	1452 (9)
40–49	2164 (13)
50–59	2736 (17)
60–69	2937 (18)
70–79	3127 (20)
80+	2676 (17)
Median (25–75%)	62 (47–75)
Gender	
Male	8406 (52)
Female	7634 (48)
Date of index diagnosis	
2000–2002	3336 (21)
2003–2005	3867 (24)
2006–2008	3962 (25)
2009–2012	4875 (30)
Index diagnosis	
Jaundice†	6951 (43)
Obstructive jaundice	2531 (16)
Cholangitis	1144 (7)
Hepatitis unspecified	408 (4)
Chronic hepatitis	541 (3)
Other liver disorders	528 (3)
Biopsy of liver	412 (3)
Any other code‡	3223 (20)
Liver test results§	
No liver test result	7354 (46)
Test results before index diagnosis	4039 (25)
Test results on or after index diagnosis	4647 (29)
HES record¶	
No HES record	3392 (21)
HES record before index diagnosis	923 (6)
HES record on or after index diagnosis	11 725 (73)
ONS mortality record**	
No ONS mortality record	10 157 (63)
Had ONS mortality record	5883 (37)

*Date of diagnosis with one of the potential cholestatic liver injury codes listed in the online supplementary material.

†Includes codes ‘Jaundice—symptom’, ‘[d]jaundice’, ‘O/e—jaundiced’, ‘[d]jaundice (not of newborn)’.

‡People in this group had an index diagnosis of any of the other codes listed in the online supplementary material.

§No liver test results=none within 90 days either side of index diagnosis date; test results before/after=closest liver test result was before/after the index and within 90 days.

¶No HES record=no HES record ever (n=1080) or no record within 365 days either side of index diagnosis date (n=2312); HES record before/after index diagnosis=closest HES record was before/after the index and within 365 days.

**ONS mortality record at any time (after index diagnosis).

CPRD, Clinical Practice Research Datalink; HES, Hospital Episodes Statistics; ONS, Office of National Statistics.

### Results

Of 16 040 cohort, 4032 (25%) were assigned as definite cases, with almost all of these assigned due to the presence of a cholestatic liver test result recorded in CPRD (table 3). None of the individuals who had ONS mortality records had ‘toxic liver disease with cholestasis’ indicated on their ONS death certificate, and after assignment of definite cases based on CPRD liver test results, HES procedure (eg, biopsy or scan) data only allowed one further definite case to be assigned. Of 16 040 cohort, 977 (6%) were assigned as possible cases of cholestatic liver injury, with the majority (947/977) due to codes related to jaundice (group 2) in both databases but a lack of liver test results. The remainder of the cohort was assigned as unlikely or non-cases.

**Table 3:** Multisource cholestatic liver injury algorithm—results of case status assignment

CPRD (Read) diagnostic code	HES diagnostic (ICD-10) code (plus HES procedural code or ONS mortality code, where considered)	CPRD liver test result	Multisource algorithm case status	(N=16 040) n (%)
Group 1|2|3*	Not considered	Cholestatic	Definite	4032 (25)
Group 1|2|3	ONS (mortality): group 1	Not considered	Definite	0 (0)
Group 1|2|3	HES biopsy/scan+group 1	Not considered	Definite	1 (0)
			Total definite	4033 (25)
Group 1	Group 1	None†	Very likely	0 (0)
			Total very likely	0 (0)
Group 1	Group 2 or no HES record‡	None	Probable	0 (0)
Group 2|3	Group 1	None	Probable	4 (0)
			Total probable	4 (0)
Group 1	Group 1|2	Not cholestatic§	Possible	1 (0)
Group 1	HES no codes of interest¶	None	Possible	25 (0)
Group 2	Group 1	Not cholestatic	Possible	4 (0)
Group 2	Group 2	None	Possible	947 (6)
			Total possible	977 (6)
Group 1	No HES record| HES no codes of interest	Not cholestatic	Unlikely	22 (0)
Group 2	No HES record| HES no codes of interest	None	Unlikely	3468 (22)
Group 3	Group 1	Not cholestatic	Unlikely	2 (0)
			Total unlikely	3492 (22)
Group 2	Group 2|no HES record| HES no codes of interest	Not cholestatic	Non-case	2869 (18)
Group 3	Group 2	None|not cholestatic	Non-case	173 (1)
Group 3	No HES record	None|not cholestatic	Non-case	340 (2)
Group 3	HES no codes of interest	None|not cholestatic	Non-case	4152 (26)
			Total non-case	7534 (47)

*Group 1=highest evidence for cholestatic liver injury and group 3=lowest evidence.

†No liver test result recorded within 90 days of index diagnosis.

‡No HES record indicates that person did not attend hospital <1 year either side of index diagnosis.

§Liver test result was recorded <90 days from index diagnosis, but results indicate either no injury or pure hepatic injury.

¶Person attended hospital <1 year from index diagnosis but no liver diagnoses of interest.

CPRD, Clinical Practice Research Datalink; HES, Hospital Episodes Statistics.

### CPRD algorithm development

#### Univariable and multivariable analysis (training cohort)

Liver test result status was shown to perfectly predict multidatabase case status, that is, all of those with CPRD cholestatic liver test results were classified as cases, while no individuals with an index diagnosis of cholaemia were classified as cases. These variables were, therefore, not considered for subsequent univariable or multivariable analysis. The univariable and multivariable results for the CPRD variables included in the final CPRD cholestatic liver injury algorithm are provided in table 4, while the univariable results for all of the potential CPRD variables initially tested and the initial fully adjusted model are provided in the online supplementary material, 8. Results for all potential CPRD explanatory variables. The two CPRD explanatory variables that were the strongest predictors of being a case were having an index diagnosis of ‘toxic liver disease with cholestasis’ (multivariable OR 20.59, 95% CI 9.41 to 45.08) or having an index diagnosis of ‘obstructive jaundice’ (multivariable OR 6.64, 95% CI 5.42 to 8.13) (table 4). Having a code for ‘jaundice’ (or similar) was also strongly associated with being a case (multivariable OR 5.10, 95% CI 4.25 to 6.11). People who had any referral recorded in CPRD within 30 days before or after the index diagnosis date were more likely to be cases (multivariable OR 1.48, 95% CI 1.33 to 1.65), as were people referred for a liver-related scan or test (multivariable OR 1.51, 95% CI 1.18 to 1.94). Having an additional liver-related diagnosis within 30 days of the index diagnosis date was also a weak predictor for being a case (multivariable OR 1.49, 95% CI 1.33 to 1.67).

**Table 4:** Descriptive, univariable and multivariable analysis of the association between being a multidatabase algorithm (definite to possible) case and the CPRD explanatory variables included in the final CPRD algorithm

CPRD explanatory variable	Total (N=8020), n (%)	Cases (N=2470), n (%)	Crude OR (95% CI)	Multivariable* OR (95% CI)	p Value†
CPRD liver test result					
None|not cholestatic	6044 (75)	494 (8)	–	–	
Cholestatic	1976 (25)	1976 (100)	–	–	
Had any referrals‡					
None	4650 (58)	1132 (24)	1	1	
One or more referrals	3370 (42)	1338 (40)	2.04 (1.86 to 2.25)	1.48 (1.33 to 1.65)	<0.001
Jaundice (or similar) index diagnosis					
No	4301 (54)	944 (22)	1	1	
Yes	3719 (46)	1526 (41)	2.47 (2.24 to 2.73)	5.10 (4.25 to 6.11)	<0.001
Cholangitis-related index diagnosis					
No	7262 (91)	2337 (33)	1	1	
Yes	758 (9)	133 (18)	0.45 (0.37 to 0.54)	1.89 (1.47 to 2.44)	<0.001
Chronic hepatitis index diagnosis					
No	7720 (96)	2464 (32)	1	1	
Yes	300 (4)	6 (2)	0.04 (0.02 to 0.10)	0.20 (0.09 to 0.45)	<0.001
Obstructive jaundice index diagnosis					
No	6774 (84)	1886 (28)	1	1	
Yes	1246 (16)	584 (47)	2.29 (2.02 to 2.59)	6.64 (5.42 to 8.13)	<0.001
Toxic liver with cholestasis index diagnosis					
No	7989 (99)	2448 (31)	1	1	
Yes	31 (1)	22 (70)	5.53 (2.54 to 12.03)	20.59 (9.41 to 45.08)	<0.001
Liver-enlargement-related index diagnosis					
No	7939 (99)	2454 (31)	1	1	
Yes	81 (1)	16 (20)	0.55 (0.32 to 0.95)	1.98 (1.12 to 3.49)	0.027
Non-specific liver-related index diagnosis					
No	7598 (95)	2439 (32)	1	1	
Yes	422 (5)	29 (7)	0.16 (0.11 to 0.23)	0.63 (0.42 to 0.95)	0.020
Number of additional liver-related diagnoses‡					
None	6165 (77)	1652 (27)	1	1	
One|more	1855 (23)	818 (44)	2.15 (1.94 to 2.40)	1.49 (1.33 to 1.67)	<0.001
Referral for liver-related scan|test‡					
No referral	7719 (96)	2312 (30)	1	1	
Had a referral	301 (4)	158 (53)	2.58 (2.05 to 3.26)	1.51 (1.18 to 1.94)	<0.001

*Multivariable OR: Frith method (see Chapter 3), adjusted for all other variables in this table. Variables were selected for inclusion in the final multivariable model by initially preparing a fully adjusted model (see online supplementary table S8), and removing those variables with p>0.05 using a backwards stepwise approach.

†p value: result of the likelihood ratio test of the association of the variable with the outcome after adjustments for all other variables in the table.

‡Multiple variables: ±30 days from index.

CPRD, Clinical Practice Research Datalink; index, index diagnosis.

##### ROC analyses of cholestatic liver injury algorithm and consideration of cut-off scores (validation cohort)

**Figure 4:**
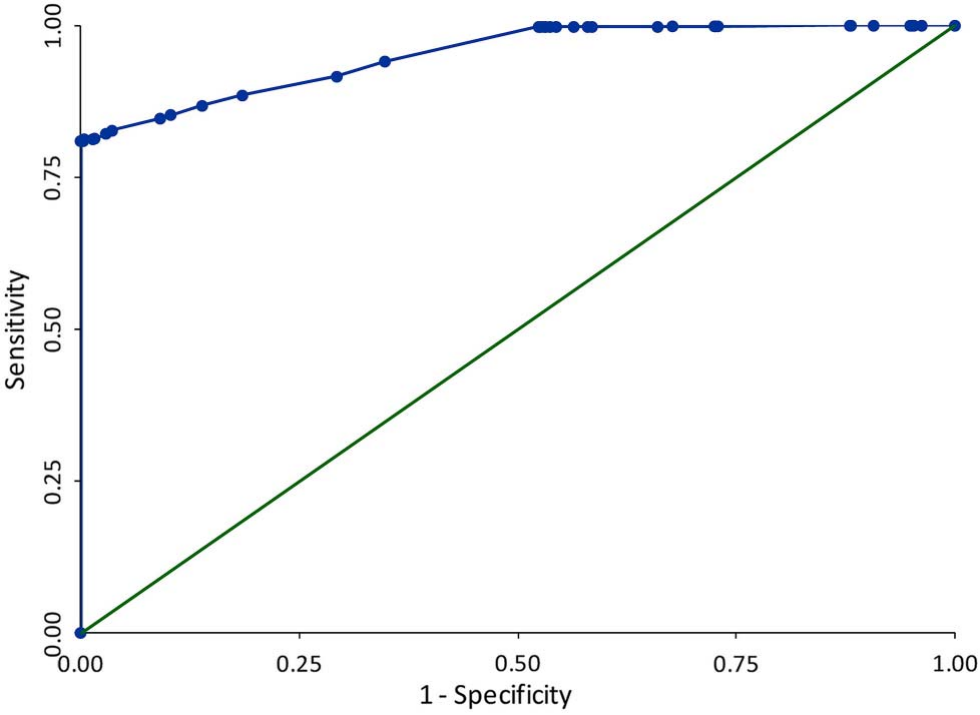
Receiver operating characteristics (ROC) graph of sensitivity against 1−specificity for a range of Clinical Practice Research Datalink (CPRD) algorithm cut-off scores, comparing the CPRD cholestatic liver injury algorithm against a multidatabase algorithm case status of probable to definite. AUC=0.95.

After adding the variables shown to be predictors of multidatabase algorithm case status in the training cohort to the validation cohort and generating a CPRD algorithm score for each person, the sensitivity and specificity of the algorithm was assessed by applying the algorithm to the validation cohort data, using a range of cut-off scores to define case status. A ROC of these results is provided in figure 4, with the full tabulation of results provided in the online supplementary material, 9. Tabulation of ROC results.

#### Area under ROC curve: 0.95

The area under the curve (AUC) value of 0.95 obtained indicates that overall the complete CPRD algorithm has an excellent ability to discriminate between individuals with a multidatabase algorithm case status of ‘definite’ to ‘possible’ and individuals with a multidatabase algorithm case status of ‘unlikely’ to ‘non-’. Tabulation of ROC results provides detail on how the sensitivity and specificity is related to the total CPRD algorithm (stage 1 and stage 2 case assignment) score; this shows that with increasing specificity, sensitivity remains high (for a specificity of 100.0%, sensitivity is over 80.0%).

## Discussion

In this study, we have compared the performance of primary care and multidatabase (primary, secondary and mortality) algorithms to identify cases of cholestatic liver injury and found that minimal additional information for case detection is provided by secondary care and mortality databases.

### Multidatabase algorithm

The results for the development of the multidatabase algorithm showed that definite case status is heavily influenced by the CPRD record, with almost all of the definite cases assigned as such based on liver enzyme level test results recorded in CPRD. ONS information did not facilitate the identification of additional definite cases, while HES data only allowed for the identification of one additional definite case.

Around half of the cohort did not have a liver test result recorded in CPRD within 90 days either side of their index diagnosis. Given that the cohort participants were selected by the presence of liver-related diagnostic codes, one could expect that standard clinical procedure would be to have performed a test of liver enzyme levels within the period of 90 days before or after the index diagnosis. Many of the individuals who did not have liver tests recorded in primary care (CPRD) are, therefore, likely to have had tests performed and recorded in secondary care. An important limiting factor of HES data is that while liver enzyme level tests are performed in UK hospitals, results are not included in the HES database. If results from hospital liver tests were available in HES, this could result in many individuals within the cohort being promoted from possible, unlikely or non-case status to ‘definite’.

The HES data in this algorithm did allow possible cases to be distinguished from unlikely cases, within people who had a code for ‘jaundice’ in CPRD but did not have any liver test results recorded. These people make up 28% of the cohort, with around a fifth of these people (6% of the cohort) identified as possible cases (rather than unlikely cases) due to the presence of a code for ‘jaundice’ in the HES data.

The lack of any information obtained from the ONS mortality data for the algorithm is likely to be due to the rarity of toxic liver disease with cholestasis as a cause of death.

### CPRD algorithm

A liver enzyme test result of cholestatic was a perfect predictor of multidatabase case status. Strong predictors were diagnostic terms that clinically would be expected to be describing a cholestatic type of liver injury (toxic liver disease with cholestasis, obstructive jaundice and jaundice). Having other referrals was also associated with being a multidatabase case.

The ROC analysis showed that the CPRD algorithm had very good ability to discriminate between the two multidatabase algorithm case statuses. This is not an unexpected result, given that the CPRD liver test results were a strong driver of the multidatabase case status (81% of the definite to possible multidatabase cases have a cholestatic liver test result in CPRD). The result does illustrate that an algorithm for identifying cholestatic liver injury that uses CPRD data alone can perform almost as well as one that uses multiple database sources.

### Implications/context

Our key finding was that for studies of cholestatic liver injury, if using linked CPRD–HES–ONS data, it is the CPRD primary care data that facilitate almost all of the ‘definite’ case status assignment. It should be noted that highly effective case-detection algorithms using data from multiple linked sources have been developed within other disease areas, for example, for vascular disease and cancer.^23^ ^24^ However, the lack of laboratory test result data is a notable deficiency of HES data that limits the added value of data linkages when working with outcomes that rely predominantly on laboratory test result data, such as cholestatic (or any type of) liver injury.

Our results do show that UK primary care (CPRD) data on its own can be used effectively for studying liver injury (and possibly other outcomes that rely on laboratory test result data). The nature of HES data meant that our goal of developing a CPRD algorithm that capitalised on the strength of linked data was somewhat limited. However, we believe that the approach that we have used in the development of a probabilistic algorithm could be of use to other researchers, particularly if validated against a superior ‘gold standard’ (such as detailed records from a specialist liver clinic). We have performed a thorough search of the literature in order to identify diagnoses and liver test criteria that informed our algorithm, and provided very clear descriptions of and/or references to these (including an internationally agreed standard for liver test criteria). We also clearly defined the time windows and data management applied for detecting the injury within CPRD. Performing a ROC analysis allowed the sensitivity and specificity of a range of algorithm scores to be presented, and case definitions corresponding to specific score cut-offs could be selected for defining cases based on different scenarios. For example, in pharmacoepidemiology, the sensitivity and specificity of the score used to identify people is likely to depend on the type of study being performed, financial resources available, time that the drug has been on the market and frequency of liver injury events associated with the drug.

Finally, an important consideration that should be applied to studies of the incidence of liver injury in CPRD (whether using just CPRD or HES-linked data) is that if one relies on laboratory test results to define a case, the estimated incidence is likely to be lower than the true population incidence, due to the group of people who have liver test results performed in secondary care but not primary care (the results of which are not currently accessible from any population-level database).

### Limitations

In the development of the CPRD algorithm, the response variable ‘case’ included multidatabase case statuses of ‘definite’ to ‘possible’. There is, therefore, a potential for people to have been incorrectly classified as cases of cholestatic liver injury in this scenario, and the development of a CPRD algorithm based on this potentially non-specific case definition could lead to the identification of false-positive cases. Including these people in the response variable ‘case’ was considered preferable to not including them, however, because it is likely that many of them did have liver tests performed in hospital that indicated cholestasis, but this information was not available within the HES database.

## Conclusions

In this article, we have attempted to capitalise on linkages between UK primary and secondary care databases in order to optimise methods for the detection of cases of cholestatic liver injury. An a priori assumption was that an algorithm that used combined information from multiple care settings (ie, including primary care data from CPRD, secondary care data from HES and ONS mortality data) would allow more accurate case identification than using primary care alone, and that this could facilitate the development of an optimised primary care algorithm. In fact, combined primary–secondary–mortality data did not strengthen liver injury case ascertainment when compared to the use of primary care data alone.
