# Supplementary material for: Optimising case detection within UK electronic health records: use of multiple linked databases for detecting liver injury
Source: BMJ Open. 2016 Sep 1;6(9):e012102. doi: 10.1136/bmjopen-2016-012102 (PMC5020862; doi:10.1136/bmjopen-2016-012102)
Supplement: supplementary material [file bmjopen-2016-012102supp_material.pdf]

Supplementary material

1. Description of data sources

CPRD contains anonymised longitudinal data on patients from over 625 NHS primary care practices from across the UK (approximately 12 million total patients) [1]. Information is recorded by general practitioners or other health centre staff as part of routine clinical care, and data quality checks at the database headquarters ensure that each practice contributing data maintains “up-to-standard” data. Data includes diagnoses (coded using standardised “Read” codes), drug prescriptions and any laboratory test results requested by GPs. A subset of CPRD-contributing practices (those in England only) have had their patient records linked to HES, which is an administrative data source that contains patient demographics, clinical diagnoses and a record of procedures performed while in hospital for every NHS hospital admission in England. CPRD-HES records linked by CPRD unique patient identifier exist from April 1997 onwards. The coding system used for diagnoses is a slight modification of the (WHO) ICD-10 standard, while procedures are identified by standard codes for hospital procedures (OPCS4 codes). ONS mortality data is linked to CPRD (and HES) via the CPRD unique patient id, and allows cause of death information (as coded on the individual’s death certificate) for people who have died to be linked to primary and secondary care data. Coding is according to the WHO ICD-10 standard.

2. Literature search for multidatabase algorithm development: details of database searched, search terms used and websites searched in order to identify publications related to the study of cholestatic liver injury in population-based databases of electronic health records (search performed on 30th June 2014)

**Medline[1] search terms**	CHOLESTASIS or DRUG-INDUCED LIVER INJURY or CHOLESTASIS, INTRAHEPATIC **and** DATABASE MANAGEMENT SYSTEMS or DATA COLLECTION or INFORMATION STORAGE AND RETRIEVAL
**Embase[2] search terms**	CHOLESTATIC HEPATITIS **and** DATA BASE
**Searches terms applied to title, abstract or keywords in both databases[3]**	CHOLESTAT* adj1 HEPAT* or CHOLANGIOL#TIC adj1 HEPAT* or CHOLESTAT* LIVER adj1(FAIL* or DISEASE* or DAMAGE*, INJUR*) or CHOLANGIOL#TIC* LIVER ADJ1(FAIL* or DISEASE* or DAMAGE*, INJUR*) **and** ADMINISTRATIVE or DATAB* or PRACTICE MANAG* adj1(SYSTEM or SOFTWARE) or AUTOMAT* adj1(RECORD*) or COMPUTER* adj7(GENERAL PRACT*) or RECORD* adj5(COMPUTER*) or ARIANNA or BIFAP or BASE DE DATOS PARA LA INVESTIGACION FARMACOEPEDEMIOLOGICA ATENCION PRIMARIA or BOSTON COLLABORATIVE DRUG SURVEILLANCE PROGRAM or BRIDGE DATABASE OF DATABASES or CALLIOPE or CCEP or CPRD or DAPI or DANISH MEDICAL REGISTRIES or EFEMERIS or ELECTRONIC MEDICAL RECORDS DATA or GECEM INVENTORY or GEPARD or GPRD or GROUP HEALTH COOPERATIVE or HOSPITAL CDM-US or HOSPITAL EPISODE STATISTICS or HES or HARVARD PILGRIM HEALTH CARE or HEALTHCORE or HENRY FORD HEALTH SYSTEMS or HEIS or HCUP or HMORN or IMS or INDIANA HEALTH INFORMATION EXCHANGE or IMPCI or INTERACTION DATABASE or IADB or INTEGRATED PRIMARY CARE INFORMATION DATABASE or KAISER PERMENENTE NORTHWEST or KAISER PERMANENTE MEDICAL CARE PROGRAM or LIFELINK or MANITOBA HEALTH RESEARCH or MEDICAID or MEDICARE or MEDICINES MONITORING UNIT or MIS or ODENSE UNIVERSITY PHARMACOEPIDEMIOLOGICAL DATABASE or OPED or PCCIU-R or PEDIANET or PEM or PHARM or PHARMO RECORD LINKAGE SYSTEM or PHARMACOEPIDEMIOLOGICAL PRESCRIPTION DATABASES OF NORTH JUTLAND or PDNJ or POPULATION DATA BC or POPULATION HEALTH RESEARCH UNIT or PRESCRIPTION CLAIMS DATA or PRESCRIPTION EVENT MONITORING DATABASE or QRESEARCH or SASKATCHEWAN HEALTH SERVICES or SEER-MEDICARE LINKED DATABASE or SIDIAP or SWEDISH CENTRE FOR EPIDEMIOLOGY or UNITED HEALTH GROUP or TAYSIDE MEDICINES MONITORING UNIT or THE HEALTH IMPROVEMENT NETWORK or THIN adj7(DATA*) or THIN adj7(COMPUTER*) or VALUE ADDED MEDICAL PRODUCTS or VACCINE SAFETY DATALINK or VETERANS ADMINISTRATIVE

	DATABASES.
**Websites searched**	The General Practice Research Database, The Health Improvement Network Database, The Hospital Episode Statistics database, The Base de datos para la Investigación Farmacoepidemiológica en Atención Primaria (BIFAP), The PHARMO institute for Drug Outcomes Research, The Boston Collaborative Drug Surveillance Program

[1]Indexed according to MeSH terms

[2]Indexed according to its own hierarchy of terms

[3] "*" indicates that any subsequent ending is acceptable, "adj" specifies the number of permissible separating words before or after the terms and "#" means any letter is permissible at this position

3. Diagnostic terms indicating liver injury

(a) Search terms used to search for relevant diagnostic terms in CPRD and HES diagnostic lists

Inclusion terms
**Search based on the word "liver"**<br>*liver* AND (*biopsy* OR *necrosis* OR *disease* OR * enlarged* OR *disorder*)
**Search based on the word "hepatic"**<br>*hepatic* AND (*failure* OR *coma* OR encephalopathy*)
**Other search terms**<br>*cholesta*, *jaundice*, *icterus*, *cholangitis*, *other gall bladder disorders*, *cholaemia*, *yellow atrophy*, *hepatitis*
**Terms excluded during search to increase specificity**
fetal, *hepatitis a*, *hepatitis b*, *hepatitis c*, *hepatitis e*, *hepatitis g*, *delive*, *pregn*, *neonat*, *perinatal*, *viral*, *virus*, *congenital*, *autoimmune*

Note 1: * represents a wildcard, which means that any text can be present in this position
Note 2: the search was set to look for words after the word "AND" on either side of the main search term (e.g. both "liver biopsy" and "biopsy liver" would be searched for)

(b) CPRD terms selected following search, grouped by strength of evidence for cholestatic liver injury

Term	*READ code*	*Strength of evidence marker*[1]
toxic liver disease with cholestasis	J635000	Group 1
[d]jaundice (not of newborn)	R024.00	Group 2
hepatitis unspecified	J633.00	Group 2
[d]jaundice	R024111	Group 2
obstructive jaundice nos	J66y600	Group 2
hepatitis unspecified nos	J633z00	Group 2
o/e – jaundiced	2274.11	Group 2
jaundice – symptom	1675.11	Group 2
[d]icterus nos	R024100	Group 2
yellow/jaundiced colour	1675.00	Group 2
o/e - jaundiced colour	2274.00	Group 2
[d]jaundice (not of newborn) nos	R024z00	Group 2
infective hepatitis	A701.11	Group 3
other liver disorders	J63..00	Group 3
chronic hepatitis	J614.00	Group 3
chronic aggressive hepatitis	J614200	Group 3
acute alcoholic hepatitis	J611.00	Group 3
other specified liver disorder nos	J63yz00	Group 3
[d]cholaemia nos	R024000	Group 3
acute hepatic failure	J600000	Group 3
o/e - liver grossly enlarged	25g4.00	Group 3
alcoholic hepatitis	J617.00	Group 3
open wedge biopsy of lesion of liver	7804200	Group 3
Cholangitis	J661.00	Group 3
biopsy of liver nec	780b000	Group 3
liver disorder nos	J63z.00	Group 3
primary sclerosing cholangitis	J661700	Group 3
chronic hepatitis nos	J614z00	Group 3
toxic hepatitis	J633000	Group 3
recurrent cholangitis	J661200	Group 3
acute hepatitis – noninfective	J600100	Group 3

Term	*READ code*	*Strength of evidence marker*[1]
o/e -liver moderately enlarged	25g3.00	Group 3
hepatic failure	J62y.13	Group 3
needle biopsy of liver nec	780a112	Group 3
toxic liver disease with chronic persistent hepatitis	J635300	Group 3
alcoholic hepatic failure	J613000	Group 3
hepatitis non a non b	A705400	Group 3
fh: hepatitis	12e3.11	Group 3
subacute hepatitis – noninfective	J601100	Group 3
encephalopathy – hepatic	J622.11	Group 3
hepatic coma	J622.00	Group 3
chronic persistent hepatitis	J614000	Group 3
calculus of bile duct with cholangitis	J646.00	Group 3
[x] hepatic failure	J625.00	Group 3
liver abscess due to cholangitis	J620100	Group 3
other specified liver disorder	J63y.00	Group 3
subacute hepatic failure	J601000	Group 3
nonspecific reactive hepatitis	J63y100	Group 3
other cholangitis	J661y00	Group 3
other non-alcoholic chronic liver disease nos	J61yz00	Group 3
ascending cholangitis	J661400	Group 3
percutaneous transvascular biopsy of lesion of liver	780a000	Group 3
toxic liver disease with hepatic necrosis	J635100	Group 3
hepatic failure as a complication of care	Sp14200	Group 3
toxic liver disease	J635.00	Group 3
toxic liver disease with chronic active hepatitis	J635500	Group 3
toxic liver disease with acute hepatitis	J635200	Group 3
acute hepatic failure due to drugs	J635700	Group 3
acute necrosis of liver	J600.00	Group 3
toxic liver disease, unspecified	J635x00	Group 3
other non-alcoholic chronic liver disease	J61y.00	Group 3
toxic liver disease with fibrosis and cirrhosis of liver	J635600	Group 3
sclerosing cholangitis unspecified	J661900	Group 3
other sequelae of chronic liver disease	J62y.00	Group 3
acute and subacute liver necrosis	J60..00	Group 3
chronic cholangitis	J661100	Group 3
recurrent hepatitis	J614300	Group 3
acute yellow atrophy	J600200	Group 3
chronic hepatitis unspecified	J614y00	Group 3
acute necrosis of liver nos	J600z00	Group 3
hepatic failure nos	J62y.11	Group 3
subacute necrosis of liver	J601.00	Group 3
cholangitis nos	J661z00	Group 3
central haemorrhagic necrosis of liver	J636.00	Group 3
toxic liver disease with chronic lobular hepatitis	J635400	Group 3
menghini needle biopsy of liver	780a111	Group 3
acute and subacute liver necrosis nos	J60z.00	Group 3
toxoplasma hepatitis	Ad05.00	Group 3
cholangitis lenta	J661500	Group 3
chronic lobular hepatitis	J614400	Group 3
subacute yellow atrophy	J601200	Group 3
subacute necrosis of liver nos	J601z00	Group 3
endoscopic ultrasound examination liver biopsy lesion liver	780f000	Group 3
obliterative cholangitis	J661600	Group 3

Term	*READ code*	*Strength of evidence marker*[1]
sheeba needle biopsy of liver	780a113	Group 3

Note 1: Group 1=strongest evidence for cholestatic liver injury, Group 2=weaker evidence for cholestatic liver injury, Group 3=weakest evidence for cholestatic liver injury

(c) HES terms selected following search, grouped by strength of evidence for cholestatic liver injury

Term	*ICD code*	*Strength of evidence marker*[1]
toxic liver disease with cholestasis	K71.0	Group 1
unspecified jaundice	R17	Group 2

Note 1: Group 1=strongest evidence for cholestatic liver injury, Group 2=weaker evidence for cholestatic liver injury

4. Classification and data management of test results

The classification of type of liver injury using test results was applied as detailed in the following table (based upon Aithal 2011[2]):

#	Type of liver injury	Liver test result
1.	Characteristic of any DILI	ALT ≥ 5 x ULN or<br>ALP ≥ 2 x ULN or<br>ALT ≥ 3 x ULN and Bil > 2 x ULN
2.	Characteristic of hepatocellular type of DILI	R* ≥ 5
3.	Characteristic of mixed type of DILI (=cholestatic hepatitis)	R > 2 and < 5
4.	Characteristic of pure cholestatic type of DILI	R ≤ 2

*R=(ALT/ULN)/(ALP/ULN), where ALT=alanine aminotransferase, ALP=alkaline phosphatase, Bil=bilirubin and ULN=upper limit of normal

In order to obtain R values from the liver test results in CPRD so that the above classification could be applied, the following data management was performed. Any results with missing ULN's had ULN values inserted based upon standard definitions of these values [3] while any identical repeated results for the same enzyme on the same day were considered as data entry errors and removed. In order for valid assessment of liver injury based on calculation of the R value, measurement of the enzyme levels should ideally be performed using the same blood sample [2]. Within the CPRD test records, blood enzyme levels recorded on the same day were considered to have been measured using the same blood sample. For days where calculation of a single R value was not possible (due to an arrangement of recorded enzyme levels other than a single ALT level and a single ALP level), data management was performed in order to obtain clean data suitable for calculation of R as shown in the following table.

Table illustrating data management of lab test results

#	Scenario	Problem	Data management performed[1]	Example illustration of change made to an individual's test record
1.	Liver enzyme level record exists but result value is empty (missing)	Cannot calculate R if no result value is present	Liver enzyme level record removed	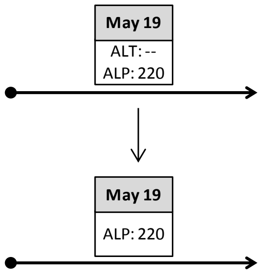
2.	Test day only has a single liver enzyme level recorded (e.g. ALT level only)	R cannot be calculated from levels for only a single enzyme type	Enzyme levels recorded <7 days of this day considered to be from the same blood sample, and record date amended accordingly[2]	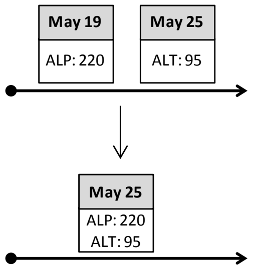
3.	Test day has ≥3 enzyme levels recorded on it, 2 of which are for the same enzyme (e.g. (1) Bil, ALP, and ALT x 2 or (2) ALP x 2 and ALT)	If there is (e.g.) 2 x ALP measurements on the same day with different values, then two possible R values can be calculated	Keep only the highest result for any duplicated enzyme level	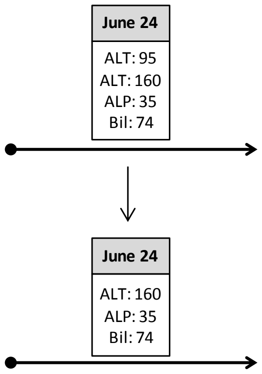
4.	Test day has 2 or 3 enzyme level records on it, and all are for the same enzyme (e.g. ALP x 2) or has 2 enzyme level records and one is Bil (e.g. Bil, ALT)	R cannot be calculated from results for a single enzyme, or from results for Bil and one other enzyme	Remove test day	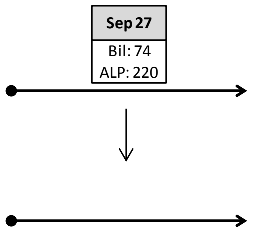

Note 1: All individuals who had test records that required any of the data management steps described here had a "data issue" variable set to "1", enabling the record to be checked during subsequent analysis if necessary

Note 2: For enzyme levels measured using the same blood sample, 7 days was considered to be the longest time that could elapse between recording the result for the first enzyme and the second enzyme in CPRD. Such a delay could be caused by administrative delay at the general practice or at the laboratory performing the tests.

5. HES procedure terms (used to identify procedures likely to elucidate the type of liver injury)

(a) Search terms used for the identification of HES procedure codes

**Inclusion terms**
liver OR *abdomen* AND (*biopsy* OR *endoscopic* OR *imaging* OR *tomography* OR *ultrasound*)

Note 1: * represents a wildcard, which means that any text can be present in this position

(b) HES procedure terms selected following search

Term	*OPCS code*
laparoscopic ultrasound examination of liver nec	J093
other specified diagnostic endoscopic examination of liver using laparoscope	J098
unspecified diagnostic endoscopic examination of liver using laparoscope	J099
biopsy of liver nec	J141
other specified endoscopic ultrasound examination of liver	J178
unspecified endoscopic ultrasound examination of liver	J179
computed tomography of abdomen nec	U081
ultrasound of abdomen	U082
magnetic resonance imaging of abdomen	U085
other specified diagnostic imaging of abdomen	U088
unspecified diagnostic imaging of abdomen	U089

6. List of CPRD algorithm potential explanatory variables

CPRD characteristic type	Potential explanatory variables (all binary[1])
Liver test	Had cholestatic CPRD liver test result
Specialist referral	Had any referrals <30 days from index date
	Had liver-related referral <30 days from index
	Top liver referral code (strength of evidence for cholestatic liver injury of referral code: Group 1 or Group 2=1, Group 3 or no referral=0))
	Referred for liver scan <30 days from index
Index diagnosis	Toxic liver disease with cholestasis (single code)
	Obstructive jaundice (single code)
	Jaundice or similar terms
	Hepatitis or similar terms
	Chronic hepatitis or similar terms
	Hepatic failure related
	Liver-enlargement related
	Cholangitis related
	Liver biopsy related
	Alcohol-related
	Liver-necrosis related
	Toxic liver disease (other than cholestasis)
	Cholaemia
	Other or non-specific code
Other liver-related diagnoses	Had additional liver-related diagnoses on index date
	Had additional liver-related diagnoses <30 days from index
	Evidence grouping for additional liver-related diagnosis <30 days from index (strength of evidence for cholestatic liver injury of additional liver-related diagnosis: Group 1 or Group 2=1, Group 3 or no additional diagnosis=0)

Note 1: The decision was made prior to analysing the data to create binary variables rather than ordered categorical (e.g. for referrals or other liver related dignoses) in order to keep the potential score variables as discrete as possible and maximise their applicability, independent of the underlying data. For example, an ordered categorical variable for referral-related information would have "No referrals" as a baseline category. If in the population being analysed everybody had a referral, no algorithm score information would be provided at all by this referral variable. Separating into binary variables means that information on the type of the referrals can still be utilised to obtain CPRD algorithm score data.

7. Increase in size of CPRD database from 2000 onwards

Year	Denominator[1]	% of current size (increase)
2000	5606488	45% (-)
2001	6263789	50% (5%)
2002	7249997	58% (8%)
2003	7886617	63% (5%)
2004	8548007	68% (5%)
2005	9048301	72% (4%)
2006	9526092	76% (4%)
2007	10097134	80% (5%)
2008	10575875	85% (4%)
2009	11066617	89% (4%)
2010	11539683	92% (4%)
2011	12075098	97% (4%)
2012	12501163	100% (3%)

[1]**Denominator:** Total number of patients registered in the database on 30th June of that year

8. Crude ORs for all potential CPRD explanatory variables and ORs for the initial fully adjusted regression model

CPRD explanatory variable		Total N = 8020 n (%)	Cases N = 2470 n (%)	Crude OR[1] (95% CI[2])	Multivariable[3] OR (95% CI)	p-value[4]
CPRD liver test result[5]	None |not cholestatic	6044 (75)	494 (8)	-	-	
	Cholestatic	1976 (25)	1976 (100)	-	-	
Had any referrals[6]	None	4650 (58)	1132 (24)	1	1	
	1 or more referrals	3370 (42)	1338 (40)	2.04 (1.86 - 2.25)	1.48 (1.31 – 1.67)	<0.001
Had liver referrals[6]	None	6513 (81)	1812 (28)	1	1	
	1 or more	1507 (19)	658 (44)	2.01 (1.79 - 2.26)	0.99 (0.66 – 1.50)	0.993
Top liver referral code[6]	None | Group 3	6936 (86)	1973 (28)	1	1	
	Group 1 or Group 2	1084 (14)	497 (46)	2.13 (1.87 - 2.43)	0.99 (0.66 - 1.49)	0.954
Hepatitis (or similar) index[7]	No	7442 (93)	2403 (33)	1	1	
	Yes	578 (7)	67 (12)	0.27 (0.21 - 0.36)	1.01 (0.05 – 19.09)	0.995
Jaundice (or similar) index	No	4301 (54)	944 (22)	1	1	
	Yes	3719 (46)	1526 (41)	2.47 (2.24 - 2.73)	4.92 (0.26 – 92.25)	0.286
Hepatic failure-related index	No	7951 (99)	2464 (32)	1	1	
	Yes	69 (1)	6 (2)	0.21 (0.09 - 0.49)	0.86 (0.04 – 17.91)	0.921
Cholangitis-related index	No	7262 (91)	2337 (32)	1	1	
	Yes	758 (9)	133 (18)	0.45 (0.37 - 0.54)	1.83 (0.10 – 34.51)	0.685
Chronic hepatitis index	No	7720 (96)	2464 (32)	1	1	
	Yes	300 (4)	6 (2)	0.04 (0.02 - 0.10)	0.19 (0.01 – 4.03)	0.290
Hepatic enceph or coma index	No	7932 (99)	2462 (31)	1	1	
	Yes	88 (1)	8 (9)	0.22 (0.11 - 0.46)	0.93 (0.05 – 18.93)	0.962
Alcohol-related index	No	7659 (96)	2423 (32)	1	1	
	Yes	361 (5)	47 (13)	0.32 (0.24 - 0.44)	1.25 (0.07 – 23.79)	0.881
Liver necrosis-related index	No	8014 (100)	2469 (31)	1	1	
	Yes	6 (0)	1 (17)	0.45 (0.05 - 3.85)	2.05 (0.06 – 64.55)	0.684
Obstructive jaundice index	No	6774 (84)	1886 (28)	1	1	
	Yes	1246 (16)	584 (47)	2.29 (2.02 - 2.59)	6.42 (0.34 – 120.42)	0.214
Toxic liver w cholestasis index	No	7989 (99)	2448 (31)	1	1	
	Yes	31 (1)	22 (70)	5.53 (2.54 - 12.03)	20.00 (0.97 – 412.64)	0.052
Toxic liver disease (non-chol) index	No	7994 (100)	2468 (31)	1	1	
	Yes	26 (0)	2 (8)	0.19 (0.04 - 0.79)	0.86 (0.03 – 21.27)	0.926
Liver-enlargement related index	No	7939 (99)	2454 (31)	1	1	
	Yes	81 (1)	16 (20)	0.55 (0.32 - 0.95)	1.91 (0.10 – 37.71)	0.669
Liver biopsy-related index	No	7689 (96)	2447 (32)	1	1	
	Yes	331 (4)	23 (7)	0.16 (0.10 - 0.25)	0.69 (0.04 – 13.27)	0.805
Cholaemia index[7]	No	8016 (100)	2470 (31)	-	-	
	Yes	4 (0)	0 (0)	-	-	
Other|non-specific liver index	No	7598 (95)	2439 (32)	1	1	
	Yes	422 (5)	29 (7)	0.16 (0.11 - 0.23)	0.61 (0.03 – 11.66)	0.741
No. of liver-related diag on index[6]	One	7734 (96)	2350 (30)	1	1	
	More than one	286 (4)	120 (42)	1.66 (1.30 - 2.10)	1.16 (0.89 – 1.50)	0.256
No. of additional liver-related diag[6]	None	6165 (77)	1652 (27)	1	1	
	One | more	1855 (23)	818 (44)	2.15 (1.94 - 2.40)	1.50 (1.25 – 180)	<0.001
Top-ranked addtl liver diag[6]	None or Group 3	6693 (83)	1842 (28)	1	1	
	Group 1 | Group 2	1327 (17)	628 (47)	2.15 (1.93 - 2.39)	0.99 (0.81 – 1.21)	0.905
Referral for liver-related scan|test[6]	No referral	7719 (96)	2312 (30)	1	1	

Had a referral	301 (4)	158 (53)	2.58 (2.05 – 3.26)	1.51 (1.18 - 1.94)	<0.001

[1]**OR:** Odds ratio, [2]**CI:** Confidence interval, [3]**Multivariable OR:** Frith method, adjusted for all other variables in the table, [4]**p-value:** result of the Likelihood Ratio Test of the assoc. of the variable with the outcome after adjustments for all other variables in the table (except CPRD liver test result and cholaemia) ,[5]**CPRD liver test result:** [6]***multiple variables:***+-30 days from index date, [6]**index:** index diagnosis. [7]**Cholaemia index**: regression analysis not performed as there were no cases with cholaemia as an index diagnosis

9. Tabulation of ROC results

CPRD algorithm score and corresponding sensitivity and specificity when comparing the CPRD algorithm against the multisource algorithm case status of definite to possible

CPRD algorithm score	Sensitivity	Specificity
-1.60	100.0%	0.0%
-1.20	100.0%	3.8%
-1.20	100.0%	4.7%
-0.81	100.0%	4.9%
-0.80	100.0%	5.1%
-0.47	100.0%	5.1%
-0.40	100.0%	9.3%
-0.07	100.0%	9.3%
-0.07	100.0%	11.9%
0.00	100.0%	12.0%
0.33	99.9%	27.1%
0.34	99.9%	27.4%
0.39	99.9%	27.5%
0.40	99.9%	32.3%
0.64	99.8%	34.0%
0.68	99.8%	41.5%
0.74	99.8%	42.0%
0.79	99.8%	42.0%
0.81	99.8%	43.3%
1.03	99.8%	43.6%
1.04	99.8%	45.6%
1.07	99.8%	46.3%
1.08	99.8%	46.8%
1.21	99.8%	46.8%
1.43	99.8%	47.0%
1.44	99.8%	47.4%
1.47	99.8%	47.4%
1.49	99.8%	47.5%
1.63	99.8%	47.6%
1.84	94.1%	65.2%
1.89	94.1%	65.2%
2.02	91.6%	70.7%
2.03	88.5%	81.5%
2.29	86.8%	86.1%
2.29	85.3%	89.7%
2.42	84.7%	90.9%
2.43	82.7%	96.4%
2.69	82.2%	97.1%
2.70	81.4%	98.4%
2.83	81.3%	98.6%
3.02	81.3%	99.6%
3.10	81.0%	99.7%
3.42	81.0%	99.9%
3.42	81.0%	100.0%
5*	81.0%	100.0%

*Score for those with a cholestatic CPRD laboratory test result (perfect predictor)

References for supplementary material

1. Herrett, E., et al., *Data Resource Profile: Clinical Practice Research Datalink (CPRD).* Int J Epidemiol, 2015. **44**(3): p. 827-36.
2. Aithal, G.P., et al., *Case Definition and Phenotype Standardization in Drug-Induced Liver Injury.* Clin Pharmacol Ther, 2011. **89**(6): p. 806-815.
3. Pagana, K.D. and T.J. Pagana, *Mosby's Manual of Diagnostic & Laboratory Tests, 4e*, in *Mosby's Manual of Diagnostic & Laboratory Tests*. 2009, Mosby.
